# Adaptation of the curriculum for the inclusion of learners with special education needs in selected primary schools in the Fort Beaufort District

**DOI:** 10.4102/ajod.v6i0.377

**Published:** 2017-10-11

**Authors:** Toyin M. Adewumi, Symphorosa Rembe, Jenny Shumba, Adeola Akinyemi

**Affiliations:** 1 School of Further and Continuing Education, Faculty of Education, University of Fort Hare, South Africa

## Abstract

**Background:**

There is need for ‘high-quality’ teachers who are equipped to meet the needs of all learners through provision of education for an inclusive society according to equal opportunities to all.

**Objective:**

This paper investigates pockets of good practice in the adaptation of the curriculum for the inclusion of learners with special education needs (SEN) in selected primary schools in the Fort Beaufort District.

**Method:**

The study adopted a qualitative research approach and employed a case study design. Eight teachers and 10 principals from 10 selected primary schools, 4 education district officials and 1 provincial official were interviewed. Purposive sampling was used to select the participants. Data were collected using document analysis and semi-structured interviews and were analysed thematically.

**Result:**

The study established that teachers use methods relating to different teaching strategies, individual work, group work and extra work.

**Conclusion:**

It was concluded that there are pockets of good practice of inclusion policy such as the use of different teaching strategies, individual work, group work and extra work for inclusion of learners with SEN in some of the selected primary schools in the poor rural context. The paper recommends adequate training for teachers in curriculum adaptation in order for all teachers to accommodate learners with SEN.

## Introduction

Post-apartheid South Africa is facing many challenges, one of which is appreciating the right to a basic education for all learners in general, and learners with special education needs (SEN) in particular. During apartheid, learners were separated and educated according to race. There were also special schools for learners with SEN (Koay et al. 2006; Walton & Lloyd 2011). To redress the segregated system and to align South Africa with other countries in accommodating learners with SEN in regular classes, South Africa has put in place a policy that encourages an inclusive education system (Al Zyoudi, Al Sartwai & Dodin 2011; Koay et al. 2006).

Inclusion is the act of educating learners who were previously educated in special schools, as a result of their special needs, in regular schools that provide the necessary support to ensure access to quality education. Curriculum adaptation refers to improvements and amendments in what is taught, methods of teaching and structures of education with the aim of accommodating all learners (Chataika et al. 2012).

The South African government’s initiative to ensure inclusive education is outlined in the 1996 Constitution and a number of policies, among them the *South African Schools Act* (SASA) of 1996 and the White Paper number 6 of 2001: Building an Inclusive Education and Training System (Department of Education 2001). The best way of reducing barriers emanating from the curriculum is to make sure the process of learning and teaching is flexible enough to involve different learning needs and styles (Department of Education 2001).

There have been concerns by media and different stakeholders that, despite implementation of inclusive education, many vulnerable learners, among them learners with SEN, remain marginalised (Ainscow 2012; Harry & Klingner 2014; Prinsloo 2001; Sisonke Consortium 2006; Tikly 2011; Walton 2011).

Informally, the public and the media have attributed the challenges encountered by children with SEN in accessing basic education to teachers using the same criteria for teaching and assessing all learners in the class, despite their diverse needs (Ainscow 2012; Motala 2011; Tikly 2011).

As a result of such prevailing conditions, learners with SEN reportedly have a high dropout rate, are often not in school at all and have poor academic performance (Ainscow 2012; Engelbrecht & Green 2007; Singh 2010; SOS Children Newsletter 2012; Tikly 2011; Walton 2011).

Many studies have been performed on the adaptation of the curriculum for the inclusion of learners with SEN but hardly any study has systematically investigated the pockets of very good practice of curriculum adaptation, especially in poor rural schools characterised by difficult working conditions and inadequate resources (Engelbrecht & Green 2007; Lomofsky & Lazarus 2001; Mastropieri & Scruggs 2005; Singh 2010; Skinner 2016). Hence, this study attempted to fill the gap by examining how the curriculum is adapted to accommodate SEN learners as well as identify other pockets of good practice.

## Research question

In this study, the researchers attempted to answer the following research question.

How is the curriculum adapted to ensure that it meets the needs of learners with SENs?

## Methodology

This paper adopted an interpretivist paradigm and the qualitative approach and used a case study design that relied largely on interviews and document analysis. The design gives in-depth details and narrative accounts from teachers, principals, education district and provincial officials on the adaptation of the curriculum for the inclusion of learners with SEN in selected primary schools in the Fort Beaufort District (Creswell 2014). The semi-structured interviews allowed the researchers to get first-hand information about the adaptation of the curriculum for the inclusion of learners with SEN in selected primary schools in the Fort Beaufort District, thus enabling them to understand the descriptions, thoughts and meanings that participants attributed to their curriculum adaptation (Kuada 2012).

This paper employed document analysis partly to fill gaps left by the interviews. Documents related to the study were revealed which included lesson plans, learners’ and teachers’ profiles. Information from these documents provided data that complemented the data gained through interviews.

Eight teachers and 10 principals from 10 selected primary schools, 4 education district officials and 1 provincial official were interviewed. Purposive sampling was used to select the participants. The gatekeepers, for instance principals and other colleagues, were employed to gain access to the research sites and participants. The data were analysed by clustering common themes, tallying stories and ranking responses to uncover the main issues that emerged. The researchers went back to ask the participants to comment on whether they felt the data had been interpreted in a manner congruent with their experiences for confirmation and verification (Creswell 2014).

## Ethical considerations

Ethical clearance was obtained from the University of Fort Hare Ethical Committees, and permission was sought from and granted by the Eastern Cape Education District. The selected participants were made to sign the informed consent forms as an indication of agreement to participate while the researchers promised to observe the code of ethics. The results of the study are discussed below. In the study, teacher, principal, education district official and provincial official participants are abbreviated as T1-T8, P1-P10, EDO1-EDO4 and PO1, respectively.

## Results

This study investigated the adaptation of the curriculum for the inclusion of learners with SEN in selected primary schools in the Fort Beaufort District. This information was sought through interviews with the selected teachers, principals, district officials and provincial official. The following are responses from teachers indicating the various methods that they used in adapting the curriculum to ensure inclusion of all learners. These manifest pockets of very good practice of inclusive education in some schools.

‘I scale it down a bit lower, so they can grasp the meaning and it can be easier for them to understand the meaning. If I see that they don’t understand Afrikaans, I have to translate the word to English for them so that at least they have the understanding of what we are working on.’ (T1, female, 48 years old)

‘If I am doing Mathematics I give the other learners difficult ones and give the slow ones the simple ones. The aim is to make sure they all understand the content. I teach all of them but if I see that they don’t understand or they are frustrated, I give them remedy. I change the methods, skills in order for them to understand the content and for me to achieve my objectives. I give them homework also in my class to ensure continuity at home. I have three groups. The first ones are the gifted learners, the second ones are the hard workers, these ones understand and the last group are my slow learners who always need my help.’ (T4, male, 53 years old)

This study also found that some of the teachers were not adapting the curriculum to meet the needs of all learners because of the size of the classes as well as a lack of training.

‘It is difficult to adapt the curriculum so that it meets the needs of all learners with full numbers of learners in the classroom. We have learners up to forty five in the class. It makes it really difficult.’ (T5, female, 37 years old)

‘Our school operates multi-grade and large classes because of shortages of teachers. Multi-grade is the combination of two or more grades in a classroom. Multi-grade, however, has its own advantage. For instance slow learners in upper grades sometimes grasp from the lesson teachers teach the lower grades.’ (T3, female, 46 years old)

T3 further said:

‘Many of the educators in public ordinary schools don’t have the skills and expertise in dealing with these learners that are experiencing barriers. We don’t have the qualification to do that.’ (T3, female, 46 years old)

These findings are evidence of good practices of inclusion of learners with SEN, particularly in the context of this study, which is within a rural, poorly resourced environment.

Most of the principals identified the various methods used by teachers in adapting the curriculum to ensure inclusion of all learners. The use of different teaching strategies, dedication of their time, group work and lowering the bar, for example giving remedial work from lower classes to accommodate all learners, were some of the methods used. Only P5 seemed not to be really familiar with what was going on in his school. The following was his comment:

‘It is sometimes difficult. The staff is basically divided into two groups; we have the experienced teachers and younger ones. Especially, the younger ones struggle to marry ideas and that is because of experience. The elderly feel more comfortable with the learners. It is difficult for me to say exactly how they adapt the curriculum. I’m not hands on in the class.’ (P5, male, 55 years old)

For their part, the education district officials in their various interviews expressed contradictory opinions. The following are some comments made in this regard:

‘Teachers adapt by using what is called curriculum adaptation but unfortunately, teachers do not do that. They come with excuses that this is extra work because curriculum adaptation is about simplifying work and giving work to learners according to their abilities. If curriculum adaptation is done in schools, learners will be fully supported in schools.’ (EDO1, male, 45 years old)

‘Teachers become impatient to start from scratch with learners. There are lots of learners who cannot read and write, we do not know where the problem is, is it with the system or the teachers?’ (EDO2, female, 45 years old)

‘We have officials with the district who are supporting the teachers. I’m not hands-on with this question but there is a friend of mine who is at the district dealing with curriculum adaptation.’ (EDO3, female, 63 years old)

‘Teachers are saying it’s difficult to implement, it is difficult for them to adapt the curriculum so that it meets the needs of the learners and with full numbers of learners in the classroom. They are having learners up to forty-five in the class, it makes it really difficult. Coming from the class myself, so it makes it difficult, I don’t think teachers are doing it the way it should be done because at the back of their minds, they have to rush, knowing that there is a learner who is lagging behind, what about the other 44 learners, that is what they are saying.’ (EDO4, female, 38 years old)

The only provincial official interviewed in this study established that the province is starting to train ordinary public school teachers on the necessary skills in identifying, assessing, adapting, differentiating curriculum and managing diversity in the classroom, through workshops. Below is his comment:

‘Educators especially at the special schools have been trained to identify and assess these learners and they also know how to do curriculum differentiation and diversity in the classroom through workshops. They know how to deal with different barriers experienced by learners. For example these teachers know how to help learners who cannot write.’ (PO1, male, 56 years old)

PO1 further said:

‘For now we are dealing with special schools and full service schools as time goes on we will roll it to the mainstream schools. We are starting to train teachers at the public ordinary schools to have necessary skills to identify and assess learners and if these learners cannot be helped they should be referred to the DBST who will refer learners to specials or full service schools.’ (PO1, male, 56 years old)

It was evident from document analysis that some of the teachers in the selected schools were actually adapting the curriculum to accommodate all learners in their classrooms. The researchers found this to be true in teachers’ lesson plans where different teaching strategies were used in the classrooms. Evidence of good practice of inclusive education policy was also found in learners’ profiles as some of these learners were given remedial work from lower classes and extra work to take home to ensure continuity.

## Discussion

This study examined the adaptation of the curriculum for the inclusion of learners with SEN in selected primary schools in the Fort Beaufort District. This study established the use of different teaching strategies, dedication of time, multi-grade, individual work, group work, extra work and coming down to learners’ level to accommodate all learners as some of the methods used to adapt the curriculum for the inclusion of learners with SEN. These findings are evidence of good practices of inclusion of learners with SEN, particularly in the context of this study, which is within a rural, poorly resourced sector. These findings are also in accordance with the rights-based approach that argues that the curriculum should be adapted to meet the needs of all learners. Education must be flexible and adaptable as there are constant changes in the challenges and needs of societies. All education systems and education programmes and campaigns must take the diversity of learners and their needs into consideration (Lohrenscheit 2002). Laurillard (2013) notes that there is no method or programme that is complete and fits all learners and teachers, and teachers have the freedom to develop their own battery of different programmes, methods, skills and knowledge to select from in making and revising curricula for individual learners and classes.

It was also found that some teachers were not adapting the curriculum to meet the needs of all learners because of the size of the classes as well as a lack of training. This finding is contrary to the rights-based approach, which states that there should be adaptation in curriculum to meet the needs of the learners with SEN (Polat 2011; Tomaševski 2004). Curriculum should be flexible and adaptable as there are constant changes in the challenges and needs of societies. The rights-based approach considers developing appropriate curriculum adaptations to match with learners’ needs instead of the learners fitting into the curriculum (Polat 2011; Tomaševski 2004). The approach places emphasis on how teachers can accommodate diversity and address SEN.

In the international literature on inclusion, emphasis is placed on strategies that ensure individual access and participation. This access is frequently obtained through creating accommodations and adaptations to teaching, learning and assessment (Alquraini & Gut 2012). Odom, Buysse and Soukakou (2011) observe that the individualised education programme is an essential component of inclusion. Differentiated instruction needs teachers to change their practices from a programme-based pedagogy to a learner-based pedagogy. Teachers endeavour to adapt pedagogical interventions to the needs of each learner, admitting that each learner varies in interests, learning profile and level of functioning. Differentiated instruction may facilitate high levels of both learner engagement and curricular achievement (Reis et al. 2011).

The study revealed that the province is starting to train ordinary public school teachers with the necessary skills in identifying, assessing, adapting, differentiating curriculum and managing diversity in the classroom through workshops. Darling-Hammond (2010) states the importance of in-service training as an essential component in the delivery of quality education, so that teachers can receive continued training in teaching methodology in order to upgrade their skills and knowledge. Richards and Rodgers (2014) state that specialist teachers are the norm internationally. They are capable of teaching a wide variety of skills and of using a range of teaching methods and strategies, so that every learner is encouraged to participate enthusiastically in teaching activities.

## Conclusion

This study revealed the various methods used by teachers in adapting the curriculum to ensure inclusion of all learners. The use of different teaching strategies, dedication of their time, individual work, group work, extra work and coming down to the learners’ level to accommodate all learners were some of the methods used. Despite the multi-grade and large classes in some of the schools, teachers were able to differentiate their teaching methods to ensure that learners with special needs are catered for. One can, therefore, conclude that the curriculum is being adapted in spite of the challenges encountered in the process, especially in schools in contexts with limited resources. There is also evidence of good practice in the fact that teacher respondents know what curriculum adaptation is and how it is supposed to be carried out. These findings are evidence of good practices of inclusion of learners with SEN, particularly in the context of this study. However, this study also found that some of the teachers were not adapting the curriculum to meet the needs of all learners because of large classes and a lack of training.

## Recommendations

From the results, the paper recommends that there be adequate training for all teachers. They should be given pre-service and in-service training to help them reorient their thinking about inclusion policy, teaching strategies, assessment modification, and adaptation of the curriculum. In addition, there is need to recruit a cohort of specialised teachers who are trained in inclusive education, so that they can influence the in-service teachers to practise inclusive programmes and change their attitudes towards learners with SEN.
